# Use of a real-life practical context changes the relationship between implicit body representations and real body measurements

**DOI:** 10.1038/s41598-021-93865-7

**Published:** 2021-07-14

**Authors:** Lize De Coster, Pablo Sánchez-Herrero, Jorge López-Moreno, Ana Tajadura-Jiménez

**Affiliations:** 1 DEI Interactive Systems Group, Department of Computer Science and Engineering, Universidad Carlos III de Madrid, Avenida de la Universidad 30, Leganés, 28911 Spain; 2 Seddi Labs, Madrid, Spain; 3 Multimodal Simulation Lab, Department of Computer Science and Architecture, Computer Systems and Languages, Statistics and Operative Investigation, Universidad Rey Juan Carlos, Madrid, Spain

**Keywords:** Psychology, Human behaviour

## Abstract

A mismatch exists between people’s mental representations of their own body and their real body measurements, which may impact general well-being and health. We investigated whether this mismatch is reduced when contextualizing body size estimation in a real-life scenario. Using a reverse correlation paradigm, we constructed unbiased, data-driven visual depictions of participants’ implicit body representations. Across three conditions—own abstract, ideal, and own concrete body—participants selected the body that looked most like their own, like the body they would like to have, or like the body they would use for online shopping. In the own concrete condition only, we found a significant correlation between perceived and real hip width, suggesting that the perceived/real body match only exists when body size estimation takes place in a practical context, although the negative correlation indicated inaccurate estimation. Further, participants who underestimated their body size or who had more negative attitudes towards their body weight showed a positive correlation between perceived and real body size in the own abstract condition. Finally, our results indicated that different body areas were implicated in the different conditions. These findings suggest that implicit body representations depend on situational and individual differences, which has clinical and practical implications.

## Introduction

The way we perceive our body has been shown to be different from what our actual body looks like, creating a mismatch between our body size/shape and the mental representations of our body (body representations)^1–4^. These distortions have been observed for both explicit (conscious body representations that are thought to be primarily constructed through visual perception of the body and cognitive-affective factors) and implicit (unconscious body representations that arise through somatosensation such as the sense of position^5^ and tactile perception^6^, proprioception, and interoception) body representations^7–9^. Furthermore, both explicit and implicit distorted self-body representations have been linked to general well-being, as well as clinical disorders such as body dysmorphia disorder and anorexia nervosa^10–14^.

A separate distinction that has been used to refer to bodily self-representations is the difference between online and offline body representations. Thus far, research has largely focused on online body representations^15,16^, defined as representations that are updated in real-time through continuous multisensory integration^17,18^, a process associated with activation in posterior parietal and premotor regions^19–23^. However, far less focus has been directed at the study of offline self-body representations, defined as abstract models of our body stored in memory that guarantee the spatial coherence of the body^18^, even though they have been shown to be important for our long-term body representations^24,25^ and are thought to code for more permanent aspects of the body such as its appearance and its motor repertoire^18^. Contrary to online body representations, representing what the body is ‘currently like’, these offline representations represent what the body is ‘usually like’ by storing long-term information about size, shape, etc.^17^.

In a recent study, offline full body representations of one’s self were investigated in a data-driven, visual, and unbiased way using a novel implementation of the reverse correlation technique^26^. This psychophysiological method has its roots in signal detection theory and auditory perception^27–29^ and has been widely used to create visual depictions of mental representations of faces^30^, sex and body shapes^31^, personal identity^32^, personality traits^33^, and racial groups^34^. Importantly, this reverse correlation technique allowed the authors to investigate implicit offline body representations (offline body representations that are accessed without explicitly asking about them), contrary to the vast amount of research that has examined these representations explicitly^1–4^. In their experiments, the authors presented female participants with a two-alternative forced choice classification task, where participants were asked to choose which of two images was most similar to their own body. These images were taken from a large set of variations of a base image of a female body by superimposing random noise patterns on this base image. Importantly, the bodies observed in the reverse correlation task are not based on participants’ real bodies. This is done intentionally, to avoid biasing the results and placing assumptions on what the participants’ mental representations of their own body should look like, and thus preserving the data-driven nature of the task as much as possible^26^. The process was repeated with a different question, where participants were asked to choose which of two images was most similar to the typical body of someone of their age and gender. The corresponding noise patterns of the selected images were then used to create two resulting classification images (CIs) that were regarded as the visualizations of the implicit mental representations of participants’ own and typical body. No correlations were observed between perceived hip width of these CIs and the real hip widths of participants (although results indicated that visual body representations of participants with negative attitudes towards their bodies showed wider hip widths than those of participants with more positive attitudes), suggesting that no relationship exists between people’s real body size and an offline, pictorial representation of their body^26^.

These findings, using the reverse correlation technique for the first time to depict implicit and offline self-representations in an unbiased and pictorial manner, are in line with previous research indicating that body size estimates are more reflective of attitudes towards one’s own body rather than real body size^35^ (but see^36^), and various studies showing that a mismatch exists between own and perceived body size^1,2,4,7,8,37–43^ (but see^44^). Thus far, research has largely focused on comparing perceived and real body measurements, although the importance of someone’s ideal body representation (internalized ideals about one’s own physical appearance^45^) and its discrepancy with actual body size has also received attention^46–49^. One study, for example, suggested that symptoms of anorexia nervosa might be related to distorted attitudes towards their ideal or desired body rather than visual distortions that lead to an overestimation of own body weight^50^. Importantly, body size estimation in these studies has been performed by framing the task of matching own and perceived body in a general, abstract way (abstract body representations; e.g. when asking whether a perceived body matches your own without framing this question within a specific context). The question arises whether the match between real and perceived body size changes when people’s body size estimation has real-life practical implications and influences real-world decision making (concrete body representations; e.g. when asking whether a perceived body matches your own while providing a practical context in which to consider this question). While research has shown that higher-level self-beliefs and attitudes^26,51,52^, as well as socio-cultural factors such as social comparison^39,53–56^ and achievement expectations and the pressures to be slim^57^, influence self-body representations in healthy populations and patients with anorexia nervosa, we suggest that higher-level beliefs about the environment might also influence self-body representations (possibly due to exchanges between these body representations and the environment through active inference^58–60^). One practical, real-life scenario, where participants are asked to judge perceived body size in relationship to a specific, well-known practical use, is provided by the online clothes shopping experience. A recent study suggests that the discrepancy between real and perceived body size persists when making an explicit choice about which model participants would use in an online clothes shopping environment^61^, even though it has been shown that these environments would greatly benefit (e.g. more purchase intentions) from an increased resemblance between the consumer and the online model/avatar^62^. The question remains whether the same is true when offline body representations are accessed implicitly.

In the current study, we employed the reverse correlation paradigm to investigate the relationship between real and perceived body size when participants had to select their own abstract (i.e. the body that looked most like their own)^26^, ideal (i.e. the body that looked most like the body they would like to have), and own concrete (i.e. the body that looked most like the body they would use for online clothes shopping) body, as well as the influence of several individual differences (bodily self-esteem, personality traits, body dissatisfaction) on these choices. Importantly, the reverse correlation technique, previously employed to construct visual depictions of participants’ own body in an abstract context^26^, allowed us to do the same with participants’ ideal body representation, as well as with the body they would use in a real-life context (i.e. own concrete body representation), using a data-driven method measuring implicit offline body representations for the first time. We hypothesized that framing own body estimation in a practical context would increase the relationship between perceived body measurements based on implicit offline body representations and real own body measurements, contrary to the absence of such a relationship when no such context is provided^26^ or when this relationship is measured explicitly^61^.

Additionally, we examined the relationship between all three implicit body representations (own abstract, ideal, and own concrete). While there is ample evidence (collected using explicit body estimation measures) for a discrepancy between own abstract and ideal perceived body representations and the role of this mismatch in body dissatisfaction^45^, the link between our own concrete (e.g. online clothes shopping) and own abstract/ideal body representations remains poorly understood. Understanding potential differences between mental representations of body size according to different contexts is important given that different neural processes might be engaged, which may have important clinical and practical implications. For example, the discrepancy between ideal and own body size estimation seems to be important in the understanding of anorexia nervosa^44^, but the relationship with body size estimation in a concrete, practical context has not yet been investigated. Furthermore, the link between own abstract, ideal, and own concrete body perception plays an important role in the design and development of online avatars for a wide range of scenarios. Evidence from 3D online game environments, for example, suggests that people prefer idealized avatars in such environments^63^. Based on this research, we hypothesized that own concrete and ideal body representations would show a stronger relationship than own concrete and own abstract body representations. Furthermore, in line with research that has shown that body size/shape estimation is affected by bodily self-esteem^26^, personality traits^61^, and body (dis)satisfaction with certain body parts^42,64^, we hypothesized that body estimation in the current study would be influenced by these psychological differences.

Finally, we aimed to explore which body areas were predictive of the different body representations (own abstract, ideal, own concrete), by using a statistical test based on random field theory specifically adapted to reverse correlation CIs^65^. This technique, which identifies clusters of pixels that predict participants’ choices, has been successfully employed to identify intensity and unpleasantness of pain expressions^66^, and facial trustworthiness^30^, for example. When it comes to body perception, researchers have used eye tracking as a method to explore the areas of the body that people look at (i.e. pay attention to) when estimating their body size. These studies have shown that people who are able to accurately determine their body size are more likely to look at informative body areas (such as the waist and upper thigh gap), while people who overestimate their body size (including women suffering from anorexia nervosa) tend to focus on less informative areas such as the face and upper parts of the torso^67^. Additionally, a recent study indicated that key areas for accurate self-assessment are located on the edges of the torso and waist on either side of the body^68^. Importantly, it seems that an equal division of attention towards both sides of the body is necessary, given that a preference for the right side of the body is associated with overestimation of own body size. While we did not use eye tracking in the current study, we wanted to explore whether similar patterns could be observed using the statistical test mentioned earlier in the paragraph, applied to all three body representations. As shown in previous research using explicit body size measures^67,68^, we hypothesized that diagnostic areas of our CIs would depend on participants’ under- and overestimation of own body measurements. Consequently, we also expected that the relationship between real and perceived hip width would be different for under- compared to over-estimators as a result of using different diagnostic areas to perform the estimation.

## Results

### Correlations between real and perceived hip width

**Figure 1:**
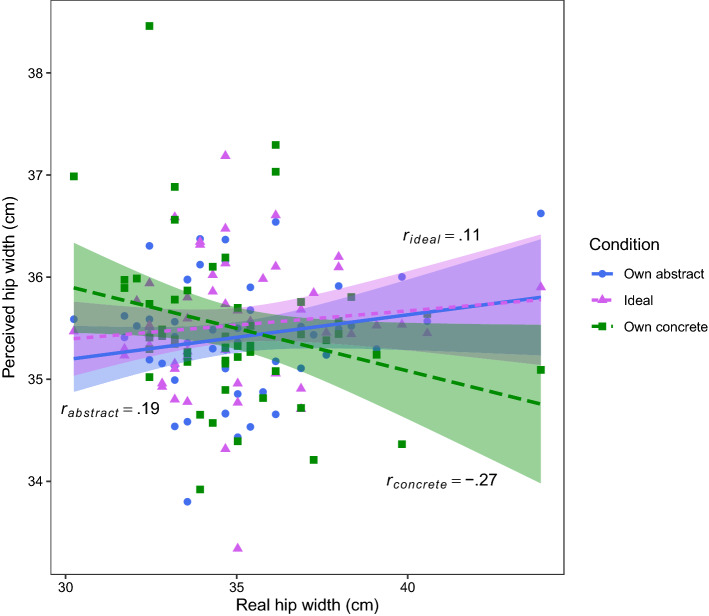
Correlations between real and perceived hip width. Perceived hip width was derived from the classification images based on participants’ responses during the reverse correlation paradigm. We correlated perceived hip width and real hip width (both in cm) for all three conditions: own abstract (*Which of these two bodies looks most like your own?*), ideal (*Which of these two bodies looks most like the body you would like to have?*), and own concrete (*Which of these two bodies looks most like the body you would use for online shopping?*). Only the latter correlation was significant.

Correlations were calculated between perceived hip width in all conditions (own abstract, ideal, own concrete) and participants’ real hip width measurements. Perceived own abstract (*r* = 0.19, *p* = 0.155, *BF*_*10*_ = 0.451) and ideal (*r* = 0.11, *p* = 0.419, *BF*_*10*_ = 0.231) hip width did not correlate with real hip width. Perceived own concrete hip width, however, showed a significant negative correlation with real hip width (*r* = -0.27, *p* = 0.050, *BF*_*10*_ = 1.09; see Fig. 1), suggesting that the hip width of CIs in the own concrete body condition was larger for participants with smaller hip widths (and vice versa). Perceived hip widths off all three conditions did not correlate with each other (own abstract—ideal: *r* = 0.08, *p* = 0.551, *BF*_*10*_ = 0.200; own abstract—own concrete: *r* = 0.02, *p* = 0.895, *BF*_*10*_ = 0.170; ideal—own concrete: *r* = -0.05, *p* = 0.690, *BF*_*10*_ = 0.182). Note that the BF for the correlation between real and perceived hip width in the own abstract condition does not meet the threshold to support the absence of any effects (although it is very close to reaching this threshold; *BF*_*10*_ < 1/3), while the BF for the own concrete condition does not meet the threshold to reject the null hypothesis (*BF*_*10*_ > 3). These findings should therefore be interpreted with more caution.

**Figure 2:**
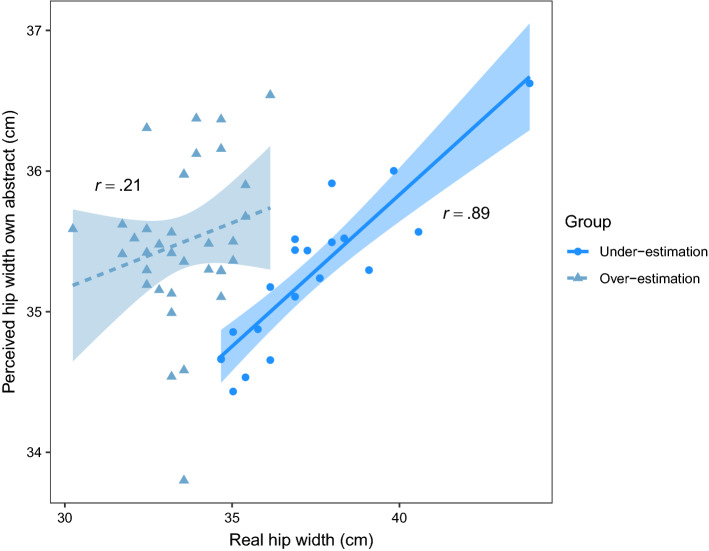
Correlations between real and perceived hip width for under- versus over-estimators. Correlation between real and perceived hip width in the own abstract condition (*Which of these two bodies looks most like your own?*), for under- (perceived hip width smaller than real hip width) versus over-estimators (perceived hip width bigger than real hip width).

In addition to the analyses across all participants, the correlation analyses were performed separately for under- versus over-estimators. Under-/over-estimators were defined as participants whose perceived hip width was smaller/larger than their real hip width. For each condition, we investigated whether the relationship between real and perceived hip width interacted with group (under- versus overestimation). For own abstract body, under-estimators showed a significantly positive correlation between real and perceived width (*n* = 20, *r* = 0.89, *p* < 0.001, *BF*_*10*_ > 100), while no relation was observed for over-estimators (*n* = 35, *r* = 0.21, *p* = 0.223, *BF*_*10*_ = 0.43; interaction with group, *F*(1,51) = 27.30, *p* < 0.001, *BF*_*10*_ > 100; see Fig. 2). No effects of under- versus overestimation were found for ideal and own concrete body.

### Psychological traits

**Figure 3:**
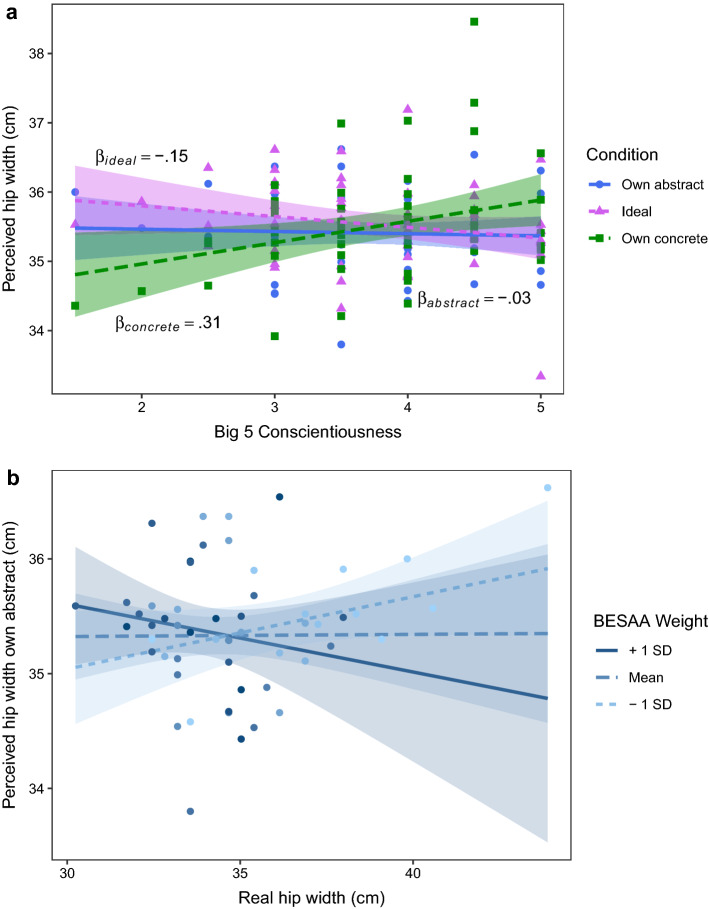
Effects of psychological traits. **(a)** A mixed linear effects model indicated that Big 5 Conscientiousness significantly predicted perceived hip width for own concrete representations (*Which of these two bodies looks most like the body you would use for online shopping?*). **(b)** A marginally significant interaction was observed between the Weight scale scores of the Body Esteem Scale for Adolescents and Adults (BESAA) and real hip width when predicting perceived hip width in the own abstract condition (*Which of these two bodies looks most like your own?*).

To investigate the influence of psychological traits on body representations, mixed linear effects models were run (see Supplementary Table S1 for model details). An H_1_ model with perceived hip width as dependent variable that included participants’ real hip width, condition (own abstract, ideal, own concrete), their interaction, and the interaction between condition and Big 5 Conscientiousness significantly improved model fit (*AIC* = 333.95, *χ*^*2*^ = 10.27, *p* = 0.016; *AIC*_*H0*_ = 357.08). Big 5 Conscientiousness significantly predicted perceived hip width for own concrete (*β* = 0.31, *SE* = 0.13, *t*(53) = 2.44, *p* = 0.018) but not own abstract (*β* = − 0.03, *SE* = 0.10, *t*(53) = − 0.34, *p* = 0.738) and ideal (*β* = − 0.15, *SE* = 0.10, *t*(53) = − 1.49, *p* = 0.143) body conditions (see Fig. 3a), indicating that participants who reported to be more conscientious showed wider perceived hip width for the own concrete body.

We additionally ran linear models with perceived hip width as dependent variable, and real hip width, psychological traits, and their interaction as predictors for each condition separately (see Supplementary Table S2 for model details). A model that included BESAA Weight scores, as well as their interaction with real hip width, significantly improved model fit for the model in the own abstract condition (*AIC* = 62.78, *χ*^*2*^ = 8.03, *p* = 0.034; *AIC*_*H0*_ = 58.30). A trending interaction was observed between BESAA Weight scores and real hip width when predicting perceived hip width for the own abstract body judgment (*β* = − 0.07, *SE* = 0.03, *t*(51) = − 1.95, *p* = 0.057; see Fig. 3b), suggesting that a negative relationship was found between real and perceived hip width for participants with more positive attitudes towards their body weight when producing own abstract body CIs, while the reverse relationship was observed for participants with more negative body weight attitudes. For the ideal and own concrete condition, no psychological trait scores significantly improved model fit.

No influence of the psychological traits was found on the differences between the body representations (own abstract—ideal, own abstract—own concrete ideal—own concrete).

### Diagnostic areas

**Figure 4:**
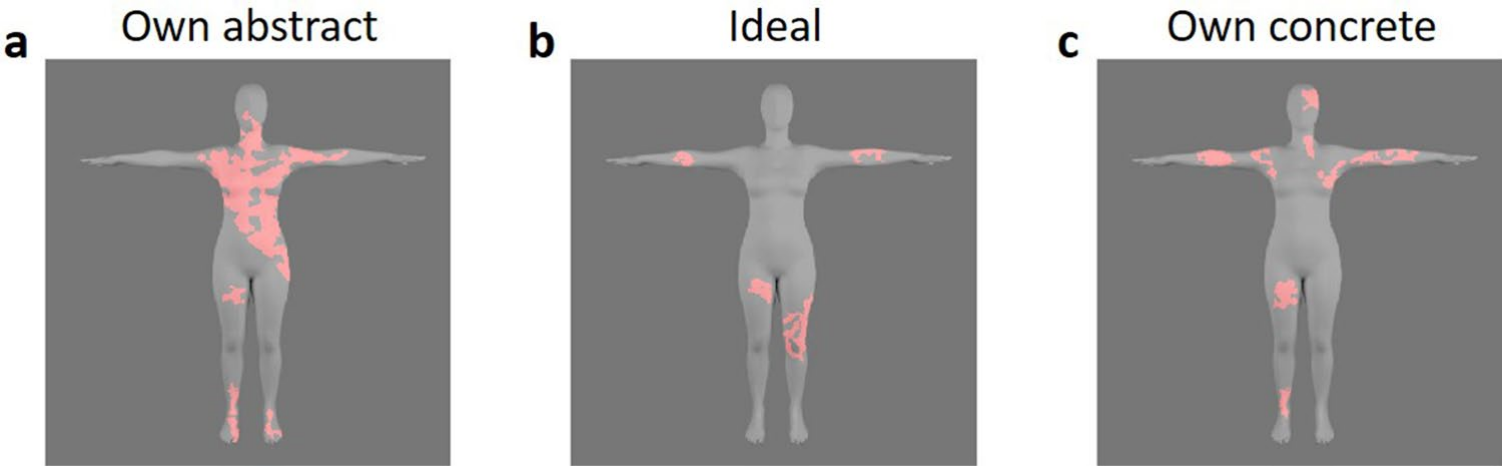
Diagnostic areas. Areas that significantly correlated with participants’ responses are indicated in red for all three conditions: **(a)** own abstract (*Which of these two bodies looks most like your own?*), **(b)** ideal (*Which of these two bodies looks most like the body you would like to have?*), and **(c)** own concrete (*Which of these two bodies looks most like the body you would use for online shopping?*). These areas identify clusters containing pixels for which luminance variation significantly predicted the different representations.

**Figure 5:**
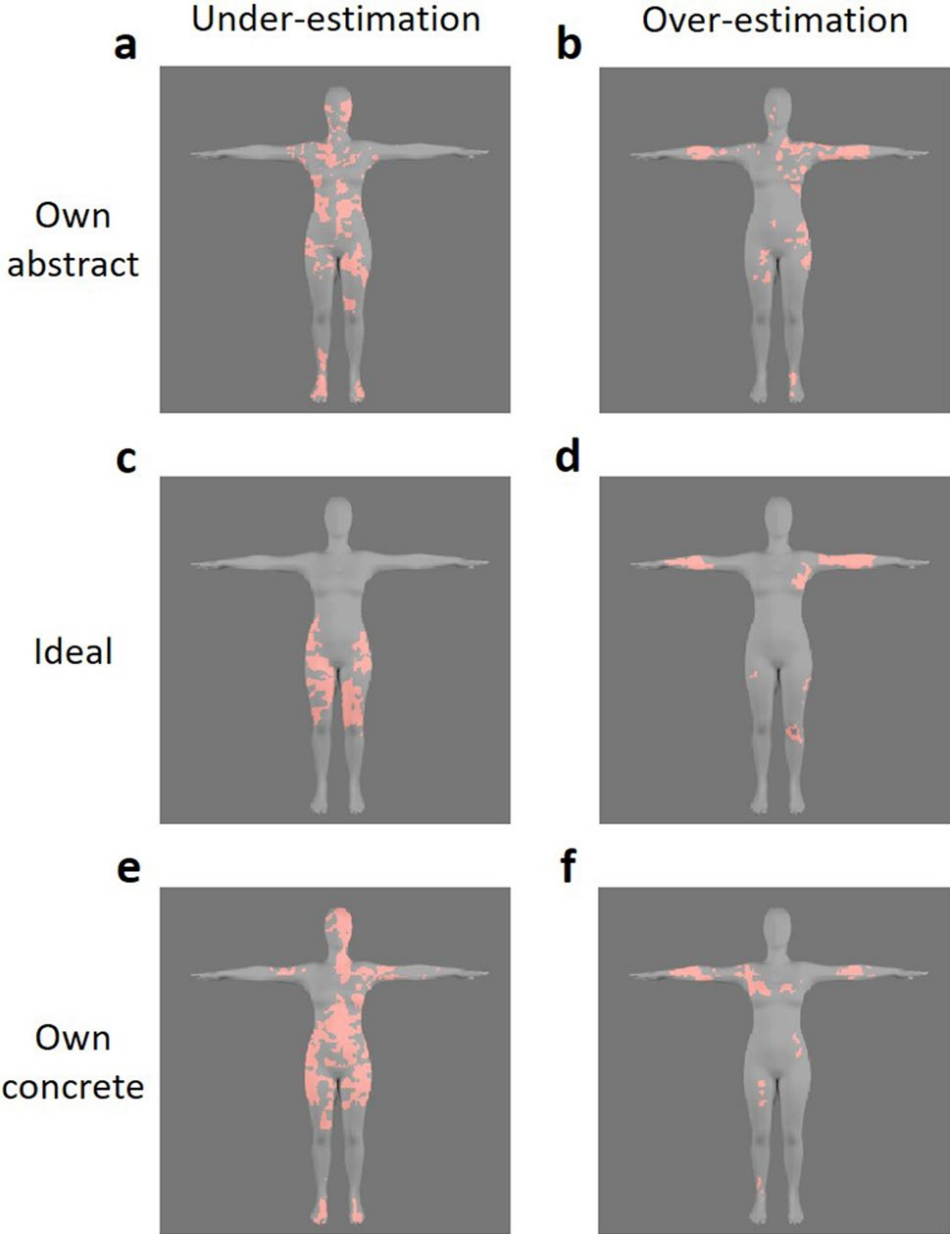
Diagnostic areas for under- versus over-estimators. Areas that significantly correlated with participants’ responses were calculated for under- (perceived hip width smaller than real hip width) and over-estimators (perceived hip width bigger than real hip width) separately. In the left-side column, areas that were significantly more predictive for under-estimators are displayed. These areas were calculated by subtracting the diagnostic areas observed in the overestimation group from those observed in the underestimation group. The reverse was done to calculate the areas that were significantly more predictive for over-estimators (right-side column). The diagnostic areas were calculated for all conditions separately: **(a,b)** for own abstract (*Which of these two bodies looks most like your own?*), **(c,d)** for ideal (*Which of these two bodies looks most like the body you would like to have?*), and **(e,f)** for own concrete (*Which of these two bodies looks most like the body you would use for online shopping?*) body representations.

All participants showed clear clusters for all three conditions. Figure 4a–c represent the CIs of the own abstract, ideal, and own concrete body respectively, across all participants. The areas in red indicate the areas that were significantly correlated with each judgment (z_crit_ ≥|2.3|, *p* < 0.05). For the own abstract body, a large cluster in the upper body, skewed to the right, was observed (including the right hip and waist, torso, neck, and right arm), as well as small clusters around the left leg and both of the feet. For the ideal body, small clusters were observed around the legs and arms. Finally, the own concrete body showed clusters located in the left leg, both of the arms, and parts of the right neck and face. A similar cluster analysis was performed by calculating the CIs across all participants who under-/over-estimated perceived compared to real hip width. The resulting images for each group were subtracted from each other for visualization purposes (e.g. underestimation–overestimation displays the areas that were uniquely significant for under-estimators). Figure 5a,c,e represent the areas that significantly predicted own abstract, ideal, and own concrete body representations for under- compared to over-estimators, while Fig. 5b,d,f depict the over- versus underestimation contrast. In the own abstract and own concrete body conditions especially, clusters distributed across the whole body significantly predicted the choices made by under- but not over-estimators.

## Discussion

In the current study, we investigated the relationship between real and perceived hip width when obtaining own abstract, ideal, and own concrete body representations using the reverse correlation technique. This method produces visual depictions of implicit body representations using a data-driven method^26,69^. First, while no correlation was found between real and perceived width when participants had to choose the body that looked most like their own (own abstract body) and the body they would like to have (ideal body), a negative relationship was observed when making a choice about which body to use for online shopping (own concrete body). However, we observed that CIs of participants who under-estimated their hip width (perceived hip width smaller than real hip width) showed a positive correlation between real and perceived hip width for own abstract body representations. Interestingly, no correlations were observed between perceived hip widths in all three conditions, which might suggest that own abstract, ideal, and own concrete body representations are represented differently. Second, our findings indicated that participants who scored higher on the conscientiousness subscale of the BFI-10 produced CIs with wider hips for own concrete body representations than less conscientious participants. Additionally, we found a positive relationship between real and perceived hip width in the own abstract condition, although only marginally significant, for participants with more negative attitudes towards their body weight, while a negative relationship was found for participants with more positive attitudes. Third, using a statistical test specifically adapted to CIs produced during reverse correlation paradigms^65^, different diagnostic areas were identified for the three body representation conditions. Interestingly, the choices that led to these body representations seemed to be predicted by areas distributed across the whole body for under- but not over-estimators.

Our findings replicate previous results showing that no correlation exists between real and perceived hip width when participants are asked to choose a body that looks most like their own in an abstract way^26^, and extend these findings by indicating that this relationship is also absent for one’s ideal body representation. Importantly, we did observe a significant correlation between real and perceived hip width for participants’ own body representations in a concrete situation. This correlation was negative, indicating that the bigger participants’ real hip width, the smaller perceived hip width of own concrete body CIs (and vice versa). This is in contrast to studies using explicit measures showing systematic under-^41^^,^^42,70^ or overestimation^1,4,71^ of perceived body size, irrespective of actual body size. Other research, however, has suggested that overestimation is a defining feature of anorexia nervosa^13^ (but see^50,72^), while underestimation is linked to obesity^37^. Our results suggest that this trend of overestimation for people with smaller bodies and underestimation for people with larger bodies is also observed in a (largely healthy) student sample for implicit body size estimation, but only when this estimation is linked to a real-life context (online shopping; although research has also found a reverse relationship for explicit body size estimation based on body mass index; BMI^9^). Furthermore, studies that have looked at body-based scaling have shown that observed objects (including avatars) are perceived relative to the size of one’s own body, rescaling optical information against internal body representations that act as a perceptual reference^73–77^. More specifically, these studies suggest that individuals who perceive their own body as large judge observed objects as smaller than they are in reality, while the reverse is true for individuals who perceive their own body as small. The current results indicate that such body-based scaling might be present when implicit body representations are framed within a practical context only, suggesting that a context with real-life implications activates specific processes. In sum, the current results show that the relationship between real and perceived body size depends on the specific context that people are asked to estimate their body size in, which has practical implications. For example, the observation of a negative relationship between real and perceived hip width for body size estimation in a practical, online context has important implications for the development and design of online avatars, as well as for online shopping in general. Research has indicated, for instance, that the impact of virtual try-ons is greatly affected by the congruency between the self and the online avatar/model, and that maximizing this perceived resemblance is a priority for the development of these online environments^62^. While idealized avatars are thought to be preferred in online contexts (driven by socio-cultural weight and body size stigmas)^63^, our results suggest that a relationship exists between perceived and real body representations in an online shopping environment, although this relationship is reversed. This seems to indicate that the influence of socio-cultural stigmas partly persists even when accessing offline body representations implicitly. It is important to note that the bodies presented to participants in the reverse correlation task are not based on their own bodies. The ability to distinguish our own from another person’s body, which mainly relies on visual perception of these bodies, is crucial for many aspects of our daily life, including social interactions^78^. Regions such as the extrastriate body area and the fusiform body area, specifically involved in own body perception, provide important self-other information at the perceptual level^79–84^, while regions such as the temporo-parietal junction^85^, medial prefrontal cortex, anterior and posterior cingulate cortex, and precuneus seem to be involved in self-other distinction at a higher level of representation^86,87^. However, research has also shown that own body representations are heavily influenced by observing the bodies of others (and vice versa^88–91^), as a result of the integration of visual information about others’ bodies into the own internal visualization of the body^92–94^, which influences self-body evaluation via social comparison^53^. Furthermore, research has shown that observing one’s own body from a third-person perspective – which makes it similar to observing others’ bodies – results in a more positive evaluation of one’s own body shape^95^ (although other research showed that first- versus third-person perspective did not influence overestimation of body part width^96^). Finally, research has also indicated that distortions in the estimation of relative body parts are present when observing both one’s own and another person’s body, indicating that our body representations influence not only the perception of our own but also the perception of others’ bodies^2^.

The absence of a relationship between all three implicit, offline body representations has implications for the underlying neurobiological mechanisms of bodily self-representations. Body perception has been shown to activate posterior parietal and premotor regions^19–23^, and interactions between these perceptual representations and affective body representations in the anterior insula and anterior cingulate cortex have been thought to lie at the basis of body (dis)satisfaction and the pathology of eating disorders^97^. However, research has primarily focused on online body representations that are continuously updated in our body schema via multisensory integration mechanisms. The question remains whether offline bodily self-representations, as investigated in the current study, activate similar neural networks and/or whether and how they interact with online representations in parietal and premotor areas. Our findings indicate that different neural processes might be employed for different types of body representations, depending on the situational context (e.g. judging own body size in an abstract context versus judging own body size for a concrete, practical use). Interestingly, it is arguable whether the ideal body representation can be considered a ‘self’ or ‘own’ body representation, given that the relationship between ideal and own body representations might not be particularly strong. Thus, such ideal body representations might be considered to be more ‘social’ or even ‘other’-based.

Importantly, our results suggest that perceived hip width of own concrete judgment CIs depended on participants’ personality traits. More specifically, CIs of participants who rated themselves as more conscientious had wider hips than less conscientious participants, irrespective of real hip width. Although previous research has indicated that conscientiousness seems to be unrelated to explicit body representations^98^, other studies suggest that conscientiousness does play a role in body representation, given that lower levels of conscientiousness are associated with higher body dissatisfaction^99^ (this relationship was marginally significant in the present study: *r* = − 0.26, *p* = 0.052, *BF*_*10*_ = 1.064). Our findings indicate that this personality factor plays a role for implicit body representations in a practical context. Although it is unclear why this effect was specific to own concrete body size estimation in our study, this suggests that implicit visual mental representations of our own abstract, ideal, and own concrete body are different from each other, and can be influenced by different factors. This is further supported by the above described absence of a correlation between perceived widths across these three body conditions, and the previously documented discrepancy between own abstract and ideal body representations^45,100^. In contrast to the influence of conscientiousness on the body representations, no correlation was observed between body (dis)satisfaction and the perceived hip widths in either of the implicit body representations, nor with the discrepancies between them (contrary to other studies^97,100^). This raises questions regarding the relationship between body (dis)satisfaction and implicit, offline bodily self-representations, and their role in the pathology of eating disorders. Further research is necessary to elucidate the neural networks associated with these offline representations, as well as their interplay with the anterior insula and anterior cingulate cortex. Importantly, however, the body (dis)satisfaction scale used in the current study was limited given that it was an explicit self-report measure using a small number of items. Future research should explore the relationship between body (dis)satisfaction and implicit, offline body representations using more comprehensive measures.

Although body (dis)satisfaction responses did not correlate with CI hip widths, a positive correlation emerged between real and perceived hip width for own abstract body representations for participants with more negative attitudes towards their body weight (as measured by the BESAA). Negative attitudes towards one’s own body have been associated with overestimation in both clinical samples such as anorexia nervosa^13^ and healthy controls^72^, although other studies report no influence of body attitudes on motor behavior driven by body representations using both explicit^101–104^ and implicit^105^ measures. Our results suggest that, independent from the accuracy of the estimation, a positive relationship between real and perceived body size when judging own body in an abstract way is present for individuals with more negative attitudes towards body weight, which may be due to different attentional biases and allocation^106–108^. Similarly, a positive correlation between real and perceived hip width for own abstract body representations was found for participants who underestimated their hip width. Interestingly, our analyses looking at diagnostic areas for the different body representations suggests that areas across the whole body were predictive for own abstract body representations within this group of participants specifically. This indicates that under- versus over-estimators focused on different body parts, and that this might have influenced the relationship between mental body representations and real body size. Under-estimators did not only seem to use areas across the whole body for own abstract, but also for own concrete body representations, while over-estimators tended to focus on isolated clusters that were primarily located on one side of the body. The latter finding is consistent with previous eye tracking research indicating that over-estimators show a bias towards one side of the body, more specifically the right upper torso/arm^68^, something which was especially apparent in the own abstract condition in the current study. Accurate estimators, however, showed a distribution of diagnostic areas that was more evenly spread onto both sides of the body, suggesting that under-estimators in the current study (who seemingly used both sides of the torso and thighs) showed a pattern of distribution similar to patterns found in accurate estimators. Furthermore, research also suggested that over-estimators tend to use less informative body areas to determine own body size (e.g. face, upper torso/arm^68^), while accurate self-assessment seems to depend on attention towards informative areas (e.g. thighs and central focus of the torso^68^, waist^67^). While our results indicate that over-estimators also focused on uninformative areas in the current study (e.g. arms), under-estimators seemed to employ a combination of both informative (e.g. waist and thighs) and uninformative areas (e.g. face) to determine their body representations when estimating their own body in both abstract and concrete ways. A focus on uninformative areas has previously been related to avoidant behavior^67^, and/or local processing (i.e. focusing on specific body parts) of visual information^109^, while a focus on informative areas such as the central part of the torso^68^ and the thighs^110^ has been linked to BMI estimation. Finally, ideal body representations for under-estimators seemed to be determined by areas around the waist and thighs across both sides of the body. This might suggest that when estimating ideal body representations, participants who under-estimate their body size distinctly use informative areas, possibly indicating the importance of these body representations^45,63^. Given the qualitative nature of these interpretations, the findings of these cluster analyses should be interpreted with caution. While more research is needed into systematic and quantitative comparisons of diagnostic areas related to different body representations, our findings were largely consistent with more time- and effort-intensive methods such as eye tracking, offering up a relatively easy and cost-effective alternative.

## Conclusion

In the current study, we explored the relationship between real and perceived body size (hip width) when asked about one’s own abstract, ideal, and own concrete body using visual depictions of implicit body representations by means of a data-driven method. In the own concrete body condition only, which referred to the body participants thought looked most like the body they would choose for online clothes shopping, we observed a negative relationship between real and perceived hip width. These results suggest that a relationship between real and perceived body measurements is only present when participants are asked to contextualize their body representations in a real-life environment. The inverse relationship, however, indicates that implicit offline full body representations, much like their explicit counterparts^1–4^, are not necessarily accurate. Furthermore, these implicit body representations were differently influenced by personality traits, attitudes towards body weight, and participants’ tendency to under- versus over-estimate their body size. Additionally, we did not observe a relationship between the three different body representations, suggesting that the implicit representation of one’s body size/shape in a practical context is not related to the own or ideal implicit body representation in abstract environments. These findings have clinical and practical implications, suggesting that the implicit mental representations of our own body depend on the situational context.

## Material and methods

### Participants

Sample size was determined following a Bayesian approach using JASP^111^. Based on previous research^26^, we initially scheduled 40 participants, and planned to check the Bayes Factor (BF; prior based on a Cauchy distribution, default scale of 0.707, zero-centered) after data collection for this group of participants. If a stopping criterion had not been reached, we would repeat this procedure after every additional five participants. The stopping criteria included: 1) the BF reached the threshold for moderate evidence to either support (BF_10_ < 1/3) or reject (BF_10_ > 3) the null hypothesis when correlating real with perceived hip width for all conditions (see below), 2) a maximum of 60 participants had been tested, 3) the end date (31/05/2020) had been reached. The experiment was terminated due to reaching the latter criterion.

55 adults (age in years: range = 18–26, *M* = 21.44, *SD* = 1.85), all female (to exclude gender differences^112^) and residing in Spain, participated in the study in exchange for a gift card of 10 euros. Participants were recruited through a local subject pool, consisting of adults taking part in research studies during the preceding year and who had agreed to be contacted to participate in future research studies. Real body measurements of all participants were available from previous studies, including weight, height, hip width, waist width, and chest width. Only participants whose real body measurements were not obtained more than six months before the start of the current study were considered. The study was conducted in accordance with the ethical standards laid down in the 1964 Declaration of Helsinki and was granted ethical approval by the local ethics committee at Universidad Carlos III de Madrid. All participants provided informed written consent beforehand. BMI in our sample ranged from 17 to 30.7 (*M* = 21.83, *SD* = 3.00), with five participants classified as underweight (BMI < 18.5), 43 as normal weight (18.5 < BMI < 24.9), five as overweight (25 < BMI < 29.9), and two participants as obese (BMI > 30). Only including participants within the normal weight range category did not change the results.

### Stimuli and apparatus

Stimuli for the reverse correlation task consisted of 400 pairs of randomly distorted images of bodies. These pairs of images were created using the ‘rcicr’ package in R^69^, which generates sinusoidal noise patterns that are superimposed on a base image. For each pair of images, a noise pattern and its inverted noise pattern are created, which distort the resulting images in perceptually opposing ways (as described in previous research^26^). All images (including the base image) were 512 × 512 pixels in size.

**Figure 6:**
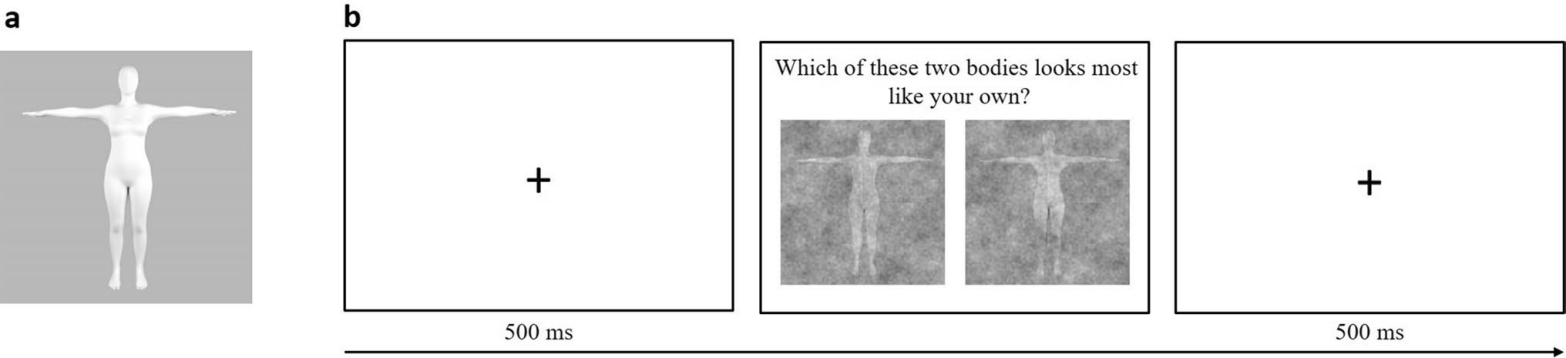
Base image and procedure. **(a)** Base image used for the reverse correlation paradigm. A sinusoidal noise pattern and its inverted noise pattern were superimposed on this base image, creating two perceptually opposing images. **(b)** Schematic overview of the experimental procedure. A fixation cross was presented for 500 ms, after which participants were asked to choose (with no time limit) between two images answering one of three questions: *Which of these two bodies looks most like your own?* (own abstract), *Which of these two bodies looks most like the body you would like to have?* (ideal), or *Which of these two bodies looks most like the body you would use for online shopping?* (own concrete).

The base image consisted of a 3D female body in a T-pose (see Fig. 6a) based on a large database of young Spanish women^113^ (10.141 women grouped into 10 ages ranges; measurements from the age group 20–24 years were used, which was the age group closest to the age range of the participants in the current study). First, a set of average measurements was extracted from this study. The most important body measurements extracted were as follows: height = 161.3 cm, chest circumference = 88 cm, waist circumference = 77 cm, hips circumference = 96.9 cm. Second, we created an avatar using skinned multi-person linear parametric modeling (SMPL)^114^, which allows for the modification of several parameters that affect the pose (e.g. moving arms and legs) and shape of the avatar (e.g. modifying fatness and height). Third, a 3D version of the measurements was created and attached to the surface of the parametric SMPL avatar, acting as body landmarks whose length changed accordingly when varying the shape parameters of the avatar. For example, by increasing the parameters usually related to fatness, a corresponding enlargement of the most relevant measurement (in this case primarily around the waist) could be observed. A custom non-linear optimization algorithm was used to automatically detect the SMPL shape parameters that defined a 3D body with measurements that were most similar to the aforementioned target measurements of the ‘average Spanish young female’. Using this method, the obtained 3D surface is a plausible estimation because the deformation space of the SMPL model is constrained to real humans, since it was learnt from variations of thousands of scanned bodies. We used the default SMPL pose values (T-pose) since it allows for the silhouette of the avatar to be observed from the front without any occlusions. The final result of this process was the closest 3D avatar mesh to the average Spanish female measurements set. To avoid bias produced by the presence of a face, the geometry in the face area was smoothed manually. Furthermore, the avatar material (matte) was chosen to avoid specular reflections. Finally, lightning was adjusted to create uniform lightning across the entire avatar body, and the background lightning was altered so that the avatar’s silhouette could be easily perceived. The avatar mesh was rendered using Maya^115^ to a 512 × 512 image.

### Psychological traits questionnaires

#### Body esteem scale for adolescents and adults (BESAA)

The BESAA is a 23-item questionnaire (Likert scale from 0 to 4), measuring people’s affective attitudes towards their bodies^116^. The questionnaire consists of three subscales: Appearance (general feelings about one’s appearance), Attribution (evaluations attributed to others about one’s body and appearance) and Weight (satisfaction with one’s body weight), with higher scores reflecting more positive attitudes.

#### Big 5 Inventory-10 (BFI-10)

The BFI-10 adapted for the Spanish-speaking community^117^ is a short form of the Big 5 Personality Test measuring Extraversion, Agreeableness, Conscientiousness, Neuroticism, and Openness using 10 items on a Likert scale from 1 to 5.

#### Body satisfaction

At the end of the questionnaire, participants were presented with three different body parts (lower, middle, and upper part of the body, with the former including the hips), as well as muscular tone and weight, and were asked to rate their (dis)satisfaction with each of these on a Likert scale from 1 to 5. The items were taken from the multidimensional body-self relations questionnaire^118^, a self-report inventory for the assessment of body image.

#### Procedure

The experiment was conducted using the online platform Gorilla^119^. Participants were sent a personal link through which they could access the experiment. In the instructions, participants were informed that the experiment had to be performed in one go (verified afterwards by the experimenter), and without outside help. The experiment consisted of three conditions. In the ‘own abstract body’ condition, participants were asked to choose *‘Which of these two bodies looks most like your own?’*. In the ‘ideal body’ and ‘own concrete body’ conditions, participants were asked to answer *‘Which of these two bodies looks most like the body you would like to have?’* and *‘Which of these two bodies looks most like the body you would use for online shopping?’* respectively. For each condition, the same 400 pairs of distorted images were used, presented in two blocks of 200 trials each (six blocks and 1200 trials total). These blocks were presented pseudo-randomly, with one block of each condition being presented in both the first three and last three blocks of the experiment. Furthermore, the third and fourth blocks of trials could not be from the same condition (i.e. blocks of the same condition could not be presented one after the other). An example of such a block order would be ‘own abstract’—‘ideal’—‘own concrete’—‘ideal’—‘own concrete’—‘own abstract’. On each trial, a 500 ms fixation cross preceded the presentation of one pair of images against a white background (see Fig. 6b). The position of the image containing the sinusoid noise pattern and its inverted counterpart was counterbalanced across trials. After participants clicked on their image of choice, the next trial started automatically. Participants were asked to answer as fast possible (no time limit was set) without responding at random. At the end of the experiment, participants filled in the questionnaires online. The experiment had a maximum total duration of 90 min.

### Data analysis

#### Correlations between real and perceived hip width

Based on participants’ responses, an average CI (weighted average of all noise patterns) was created for each participant for each condition (own abstract, ideal, own concrete) using the ‘rcicr’ package^69^. Perceived hip width (as an index of body size particularly associated with body dissatisfaction^41^) of these CIs was calculated as described in previous research^26^ using a psychometric curve^120^. First, we symmetrically defined two 10 × 26 regions of interest (ROIs) around the left and right hip. After normalization of the greyscale pixel values (darkest pixel = 0, lightest pixel = 1), we fit the psychometric function to the luminance change of the pixels in each ROI and calculated the point of subjective equality (PSE) as the pixel on the horizontal axis that corresponded to the mid-point of the vertical axis. These pixel values were considered estimates of the edge location of each hip. The left pixel was subsequently subtracted from the right pixel, and this value was converted to hip width in cm using a scaling factor (0.3623). After this was done for each row within the ROIs, we averaged the values across rows. No outliers were detected (perceived hip width ≥ 2.5 standard deviations from the mean in either condition). Finally, this obtained perceived hip width was correlated with participants’ real hip width using simple Pearson’s *r* in R^121^. We corrected for multiple comparisons using false discovery rate correction and normality checks were performed with Shapiro-Wilks tests (all *p*s > 0.314). In addition to a frequentist analysis, we performed a Bayesian analysis^122^ using JASP^111^. The Bayesian approach was used to test 1) whether there was moderate to strong evidence to reject the null hypothesis under a Bayesian framework in case of a significant effect, and 2) whether potential null results could be considered support for the absence of any effects. We calculated the Bayes Factor (BF_10_), representing the observation of our data under the alternative hypothesis (H_1_) compared to the null hypothesis (H_0_), and employed a threshold of moderate evidence to support (BF_10_ < 1/3) or reject (BF_10_ > 3) the null hypothesis.

#### Psychological traits

To investigate the influence of psychological traits (BESAA, BFI-10, body satisfaction) on perceived hip width, mixed linear effects models were used with perceived hip width as the dependent variable^26^. First, an H_0_ model was calculated including three predictors: participants’ real hip width, condition (own abstract, ideal, own concrete), and their interaction. Subsequently, using a systematic model comparison procedure, the psychological trait scores were entered into the model to verify which trait score(s) significantly improved model fit (H_1_). Psychological (sub)-trait scores were entered into the model one by one, and were included in the model if they significantly improved model fit as indexed by the Akaike Information Criterion (AIC). Additionally, for each condition separately, we examined whether the relationship between perceived and real hip width was moderated by the psychological traits by employing a linear model with perceived hip width as dependent variable and real hip width as its predictor (H_0_). Subsequently, the psychological trait scores and their interaction with real hip width were entered into the model (using the model comparison procedure as described above) to verify which of the predictor terms significantly improved model fit (H_1_).

#### Diagnostic areas

Own abstract, ideal, and own concrete body CIs were calculated across participants, after which the ‘stat4CI’ toolbox^65^ was used to perform a cluster test on each CI at the group level to identify the areas that were significantly correlated with participants’ responses. We first smoothed the CI noise pattern using a Gaussian filter (σ_b_ = 5). The smoothed image was masked by the base image and *z*-transformed. A two-tailed cluster test (z_crit_ ≥|2.3|, *p* < 0.05) was performed to identify clusters containing pixels for which luminance variation predicted own abstract, ideal, and own concrete body representations. Importantly, the findings of these cluster analyses should be interpreted with caution given that their interpretation is qualitative rather than quantitative in nature, and that statistical comparisons between conditions are impossible. For this reason, we focused our interpretation of these results on areas that were highlighted in previous literature that showed that healthy and clinical populations are often dissatisfied^123^ with regions such as the torso^124,125^, as well as stomach, waist, hips, and thigh regions^42,126^.

## Supplementary Information

Supplementary Dataset.

Supplementary Table S1.

Supplementary Table S2.

## Data Availability

All data is available in the Supplementary Material.
