# Supplementary material for: Use of a real-life practical context changes the relationship between implicit body representations and real body measurements
Source: Sci Rep. 2021 Jul 14;11:14451. doi: 10.1038/s41598-021-93865-7 (PMC8280174; doi:10.1038/s41598-021-93865-7)
Supplement: Supplementary file 2 — Supplementary Table S1. [file 41598_2021_93865_MOESM2_ESM.docx]

*Supplementary Table S1. Model parameters (fixed effects) included in the winning model of the mixed linear effects model to investigate the influence of psychological traits on body representations.*

Dependent variable = perceived hip width. For Condition, the reference category is ‘own abstract’. * = *p* ≤ .05.

| Predictor | Estimate | Confidence interval | *p*-value |
| --- | --- | --- | --- |
| Big 5 Conscientiousness | -.03 | -.24 – .18 | .769 |
| Condition: Ideal | 0.58 | -.55 – 1.72 | .321 |
| Condition: Own concrete | -1.18 | -2.32 – -.05 | .050* |
| Big 5 Conscientiousness: Ideal | -.12 | -.42 – .17 | .422 |
| Big 5 Conscientiousness: Own concrete | .34 | -.05 – .63 | .027* |
