# Supplementary material for: Use of a real-life practical context changes the relationship between implicit body representations and real body measurements
Source: Sci Rep. 2021 Jul 14;11:14451. doi: 10.1038/s41598-021-93865-7 (PMC8280174; doi:10.1038/s41598-021-93865-7)
Supplement: Supplementary file 3 — Supplementary Table S2. [file 41598_2021_93865_MOESM3_ESM.docx]

*Supplementary Table S2. Model parameters included in the winning model of the linear model to investigate whether the relationship between perceived and real hip width was moderated by the psychological traits.*

Dependent variable = perceived hip width in the ‘own abstract’ condition. BESAA = Body-Esteem Scale for Adolescents and Adults. * = *p* ≤ .05, † = *p* ≤ .10.

| Predictor | Estimate | Confidence interval | *p*-value |
| --- | --- | --- | --- |
| BESAA Weight | 2.32 | -.09 – 4.73 | .059† |
| Condition: Ideal | 0.17 | .02 – .31 | .026* |
| Condition: Own concrete | -.07 | -.14 – .00 | .057† |
